# Multiple Subchondral Bone Cysts Cause Deterioration of Articular Cartilage in Medial OA of Knee: A 3D Simulation Study

**DOI:** 10.3389/fbioe.2020.573938

**Published:** 2020-10-06

**Authors:** Adeel Anwar, Zhenwei Hu, Yufang Zhang, Yanming Gao, Cong Tian, Xiuying Wang, Muhammad Umar Nazir, Yanfeng Wang, Zhi Zhao, Decheng Lv, Zhen Zhang, Hu Zhang, Gang Lv

**Affiliations:** ^1^Institute of Translational Medicine, China Medical University, Shenyang, China; ^2^Department of Orthopaedic Surgery, The Second Hospital of Chaoyang City, Chaoyang, China; ^3^Department of Mechanical Engineering, Dalian Jiaotong University, Dalian, China; ^4^Department of Orthopaedic Surgery, The Second Affiliated Hospital of Dalian Medical University, Dalian, China; ^5^Department of Railway Vehicle, Ji Lin Railway Technology College, Ji Lin, China; ^6^COMAC, Beijing Aircraft Technology Research Institute, Beijing, China; ^7^Department of Anesthesia, The Second Affiliated Hospital of Dalian Medical University, Dalian, China; ^8^Department of Orthopaedic Surgery, The First Affiliated Hospital of China Medical University, Shenyang, China; ^9^Department of Orthopaedic Surgery, The First Affiliated Hospital of Dalian Medical University, Dalian, China; ^10^Department of Orthopaedic Surgery, The 920th Hospital of Joint Logistics Support Force, Kunming, China

**Keywords:** osteoarthritis, knee, subchondral bone cysts, cartilage degeneration, finite element analysis

## Abstract

**Aims:**

To investigate the impact of subchondral bone cysts (SBCs) in stress-induced osseous and articular variations in cystic and non-cystic knee models using finite element analysis.

**Materials and Methods:**

3D knee joint models were reconstructed from computed tomography (CT) and magnetic resonance imaging (MRI). Duplicate 3D models were also created with a 3D sphere mimicking SBCs in medial tibia. Models were divided into three groups. In group A, a non-cystic knee model was used, whereas in groups B and C, SBCs of 4 and 12 mm size were simulated, respectively. Cyst groups were further divided into three sub-groups. Each of sub-group 1 was composed of a solitary SBC in the anterior half of tibia adjacent to joint line. In sub-group 2, a solitary cyst was modeled at a lower-joint location, and in sub-group 3, two SBCs were used. All models were vertically loaded with weights representing double- and single-leg stances.

**Results:**

During single-leg stance, increase in subchondral bone stress in sub-groups B-1 and B-3 were significant (*p* = 0.044, *p* = 0.026). However, in sub-group B-2, a slight increase was observed than non-cystic knee model (9.93 ± 1.94 vs. 9.35 ± 1.85; *p* = 0.254). All the sub-groups in group C showed significantly increased articular stress (*p* < 0.001). Conversely, a prominent increase in peri-cystic cancellous bone stress was produced by SBCs in groups B and C (*p* < 0.001). Mean cartilage shear stress in sub-groups B-1 and B-2 (0.66 ± 0.56, 0.58 ± 0.54) was non-significant (*p* = 0.374, *p* = 0.590) as compared to non-cystic model (0.47 ± 0.67). But paired cysts of the same size (B-3) produced a mean stress of 0.98 ± 0.49 in affected cartilage (*p* = 0.011). Models containing 12 mm SBCs experienced a significant increase in cartilage stress (*p* = 0.001, *p* = 0.006, *p* < 0.001) in sub-groups C-1, C-2, and C-3 (1.25 ± 0.69, 1.01 ± 0.54, and 1.26 ± 0.59), respectively.

**Conclusion:**

The presence of large-sized SBCs produced an increased focal stress effect in articular cartilage. Multiple cysts further deteriorate the condition by increased osseous stress effect and high tendency of peripheral cyst expansion in simulated cystic knee models than non-cystic knee models.

## Introduction

Osteoarthritis (OA) of the knee joint is a common degenerative joint disease. The advanced cases of OA are usually associated with formation of subchondral bone cysts (SBCs) (Ondrouch, 1963). Nearly 50% of knee OA cases present with SBCs (Wu et al., 2007; Raynauld et al., 2008). In the majority of cases, these are spherical or ellipsoidal in shape and located at the articular surface. These are thought to be specific bony adaptations in the weight-bearing areas of joints (Crema et al., 2010; Javaid et al., 2010). Two different theories (synovial intrusion theory and bony contusion theory) described the formation of SBCs in weight-bearing regions (Freund, 1940; Landells, 1953; Rhaney and Lamb, 1955; Ondrouch, 1963). However, more recently the concept of vascular pathology (hypertension) has been proposed about the formation of SBCs in non-load bearing regions (Chan et al., 2017).

Though there is no consensus about the exact relationship between SBCs and knee pain, it is widely accepted and several recent studies have argued that there is an association between SBCs and knee pain in OA knee patients (Landells, 1953; Kornaat et al., 2006; Torres et al., 2006; Javaid et al., 2012). In clinical practice, X-rays remain the key investigation for the diagnosis of osteoarthritis. Advanced imaging modalities, such as CT and MRI, show more accurate and precise structural morphology of these cystic lesions (Marra et al., 2008; Roemer et al., 2009; Crema et al., 2010). In a clinical study using MRI, Raynauld et al. (2008) found the correlation between change in mean cyst size in millimeters and loss of medial femoral condyle cartilage in individuals with knee osteoarthritis. The exact relationship between bone marrow lesions (BMLs) and SBCs is not clear; however, some recent studies have advocated that BMLs may develop into SBCs (Carrino et al., 2006; Crema et al., 2008, 2010).

On the other hand, biomechanical studies addressed stress changes in the osseous elements (Durr et al., 2004; McErlain et al., 2011). A 2D finite element (FE) study demonstrated that the SBCs produce stress effect and stress-induced micro-fractures in the femoral head. The authors hypothesized that these micro-fractures might be the first step in the development of SBCs in OA (Durr et al., 2004). Another study advocated that SBCs produce increased osseous stress around the cyst (McErlain et al., 2011). To our best knowledge, all of these studies have focused on stress changes in osseous elements only while ignoring subchondral bone and articular cartilage degeneration (Durr et al., 2004; McErlain et al., 2011).

The detailed effect of multiple cysts on the knee joint is lacking in the literature. Moreover, a comparison between a single cyst and multiple cysts in relation to cyst location has not been described before. It is meaningful to investigate associations between subchondral bone cyst parameters (e.g., number, size, and location) and associated articular degeneration in OA pathology. Finite element analysis is well known and widely accepted in orthopedics (Bosiakov et al., 2017; Trad et al., 2018; Bini et al., 2019). In this technique, 3D models are used with simulated loads to analyze and predict the outcomes. The aims of this simulation study were to (a) establish the biomechanical stress-strain profile of different-sized SBCs in tibia; (b) evaluate any correlations between cyst number, size, position, and quantitative stress changes; and (c) determine intra-osseous, intra-cystic compression variations and the resultant stress changes in subchondral bone and articular cartilage using the non-cystic and cystic 3D knee models. The results of this simulation study provide insight into the role of SBCs on biomechanical aspect of articular degeneration in OA pathogenesis.

## Materials and Methods

### Three-Dimensional Modeling

**FIGURE 1:**
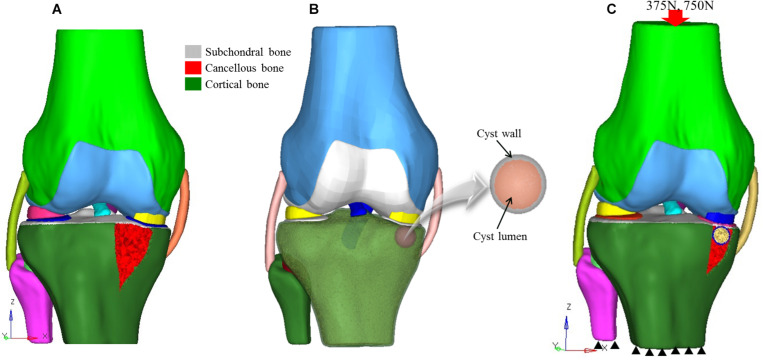
3D knee models. **(A)** Non-cystic 3D knee model showing different structures, **(B)** model of SBC knee model, and **(C)** finite element model showing loading and boundary conditions. SBC, subchondral bone cyst.

**FIGURE 2:**
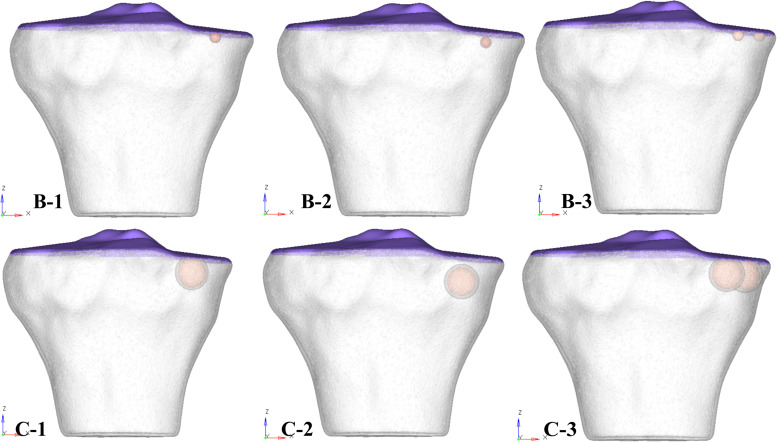
3D tibial plateau models showing size, number, and location of SBCs in three sub-groups of group B and group C. SBCs, subchondral bone cysts.

Institutional ethical approval was obtained before this study, and it was performed according to the Declaration of Helsinki. CT and MR images of an individual (52 years old, male) with early medial compartment knee OA were used for modeling. There was no past history of trauma. The geometrically accurate three-dimensional (3D) bony model of the knee joint was generated from CT scan images of the right knee in the neutral unloaded position. The 1.5 mm slice thickness CT DICOM images were imported into Mimics 10.1 software (Materialise, Leuven, Belgium) to reconstruct the surface geometry of the femur, tibia, and fibula. MR images were used for the reconstruction and reference points of soft tissues including the ligaments (ACL, PCL, MCL, and LCL), menisci and cartilaginous components of the distal femur, tibial plateau, and tibio-fibular joint (TFJ). In Geomagics Studio 11.0 software (Raindrop Company, United States) IGS files of the bony elements were used to get individual structure’s volumetric form. Soft tissues were also handled and assembled in Geomagics. These models were then assembled and exported as STP files representing the 3D knee complex as shown in Figure 1A. The coordinate axes of the assembled models were aligned as the *X* axis pointed medially (lateral to medial femoral condyle), the *Y* axis pointed posteriorly (anterior to posterior), and the *Z* axis pointed upward (knee to hip). To simulate the SBCs of different size, number, and location, computer-aided design (CAD) software (ProE CREO 3.0 PTC Corp., United States) was used. The 3D model of SBC is depicted in Figure 1B. To illustrate the exact fit SBC-bone model, there was no gap between bone and cyst capsule. The bone cysts were modeled with spherical morphology. Two different-sized (diameters: 4 and 12 mm), single and paired cysts located in upper and slightly lower position in relation to joint line were simulated (see Figure 2 and Table 2 for cysts parameters). In total, seven knee models were simulated in this study. All the models were meshed using the software HyperMesh 14.0 (Altair Engineering, Inc., United States). Based on mesh convergence study, the mesh size of 0.3 mm for bones, soft tissues (ligaments, menisci, cartilages) and SBCs was used. The type of mesh used in this study was quadratic tetrahedral elements to minimize elements stiffness. The cortical, cancellous, and subchondral components of bones were established as shown in Figure 1A.

### Finite Element Modeling and Material Properties

The reconstructed 3D models (ING files) were imported into finite element analysis software Abaqus 6.14 (Simulia Corp., United States). Interactions were carried out between different parts of the models using this software. The cortical, subchondral, and cancellous bony portions were assigned the Young’s modulus (*E*) of 7,300, 3,000, and 1,100 MPa and Poisson ratio (υ) of 0.3, 0.3, and 0.26, respectively (Choi et al., 1990; Kim et al., 2011; Qiu et al., 2011; Raja Izaham et al., 2012). FE models of soft tissues included cartilage, menisci, and major ligaments (cruciate and collateral ligaments). The menisci and cartilage were modeled as linear-elastic materials (Donahue et al., 2002). Articular cartilage was defined as Young’s modulus of 12 MPa and Poisson’s ratio of 0.45, whereas menisci were modeled with Young’s modulus of 80 MPa and Poisson’s ratio of 0.3 (Hopkins et al., 2010). Interfaces between the cartilage and bones were fully bonded. Contact was assigned between the femoral cartilage and meniscus, between meniscus and tibial cartilage, and between femoral and tibial cartilage on both the medial and lateral sides. Surface friction coefficient of 0.02 was used, which is in the normal range for human articular joints (Mow et al., 1993). The ligament models were defined as rubber-like materials with stress-strain relationship according to previous studies (Takeda et al., 1994; Mesfar and Shirazi-Adl, 2005; Innocenti et al., 2009; Innocenti et al., 2014; Koh et al., 2019).

### Validation of 3D Model and Boundary Conditions

A review of the literature shows that in the majority of SBCs cases, a secondary closure is more commonly found than an opening (Landells, 1953). Therefore, we simulated a complete cyst model without any opening at the joint surface. The 3D osseous and soft tissues models and material properties applied in this experiment were assigned according to the previous published studies (see Table 1). The simulated force was applied proximally to the femur, whereas the distal end of the tibia was fixed in all degrees of freedom (McErlain et al., 2011). All the models were tested for two different physiological conditions, i.e., two-leg and one-leg standing. The individual’s body weight was 75 kg in this study; therefore, about 50% (375 N) of the body weight was applied to simulate two-leg standing condition and a load of 750 N for single-leg standing in axial direction, as given in Figure 1C.

**TABLE 1:** Validation of material properties used in this study with literature.

Model	Young’s modulus (MPa)	Poisson’s ratio	References
Cortical bone	7,300	0.3	Kim et al., 2011; Qiu et al., 2011
Subchondral bone	3,000	0.3	Choi et al., 1990
Cancellous bone	1,100	0.26	Kim et al., 2011; Qiu et al., 2011
Cartilage	12	00.45	Hopkins et al., 2010
Menisci	80	0.3	Hopkins et al., 2010
Anterior cruciate ligament (ACL)	169	0.45	Innocenti et al., 2016
Posterior cruciate ligament (PCL)	177	0.45	Innocenti et al., 2016
Medial collateral ligament (MCL)	332	0.45	Innocenti et al., 2016
Lateral collateral ligament (LCL)	345	0.45	Innocenti et al., 2016
Cyst	12	0.45	Sarrafpour et al., 2019

### Grouping

For the ease of results interpretation, models were categorized into three groups. Group A consisted of non-cystic knee model. Group B and group C were composed of SBCs knee models of 4 and 12 mm (diameters), respectively (data from clinical series given in Supplementary Table 1; the smallest SBC was 4 mm and largest 12 mm). We further simulated single and multiple cysts, of different locations (upper and lower) in respect to articular surface, as well as anterior and posterior halves of the medial tibial plateau. Groups B and C were further subdivided into three sub-groups: 1, 2, and 3. In sub-group 1, one cyst was present in the anterior half of medial tibial plateau adjacent to the joint line (Figure 2, represented by B-1 and C-1). In sub-group 2, a solitary cyst was modeled in the lower location of anterior half of medial tibia (∼4 mm from joint line) (Figure 2, represented by B-2 and C-2). Sub-group 3 demonstrated two cysts, one in anterior and another in posterior tibial half (Figure 2, represented by B-3 and C-3).

### Statistical Analysis

The statistical analysis was done using SPSS 16.0 (SPSS Inc., Chicago IL). Descriptive statistics was used to determine means and standard deviations. One-way ANOVA and multiple comparison least significant difference (LSD) tests were used to determine the mean difference. *p*-value was considered significant when *p* < 0.05. Nodal analysis was done in the region of interest (ROI), using selected nodes (*N* = 20) from each sub-group of 3D model. Von Mises stress (VMS), Tresca stress, and cartilage shear stress distributions in the subchondral bones, the cancellous bones, and cartilage were analyzed. Strain analysis of the SBCs was also calculated.

## Results

The total number of nodes and elements in the non-cystic knee model were 1,034,891 and 4,911,748 respectively, whereas the total number of nodes in solitary small cyst model were 1,488,268 and 1,488,268 elements. The knee model with solitary larger-sized SBC was composed of 1,504,518 nodes and 1,504,518 elements. The detail of individual 3D models is given in Table 2. The effect of SBCs on bony elements (subchondral and cancellous) and articular cartilage was compared with the stress changes in the non-cystic knee model.

**TABLE 2:** Detail of SBCs parameters and nodes and elements in different models.

Serial no.	Experimental models	SBC size, diameter	Cyst co-ordination			Total no. of nodes	Total no. of elements	FE in cysts *N*: Nodes *E*: Elements
			Distance b/w cyst center and cartilage	Distance b/w cyst center and lateral bone	Distance b/w cyst centers			
1	Group A: Non-cystic knee model					1,034,891	4,911,748	
2	Group B-1: Solitary, smaller SBC near the joint line	4 mm	2.92 mm	2.99 mm		1,488,268	1,488,268	*N* = 12,675
3	Group B-2: Solitary, smaller SBC apart from joint line	4 mm	4.54 mm	2.86 mm		1,488,434	7,349,333	*E* = 50,146
4	Group B-3: Paired, smaller SBCs model	4 mm, 4 mm	Ant: 2.92 mm Post: 2.98 mm	Ant: 3.0 mm Post: 3.07 mm	11.64 mm	1,490,094	7,356,280	*N* = 12,785
5	Group C-1: Solitary, larger SBC model near the joint line	12 mm	7.22 mm	7.55 mm		1,504,518	1,504,518	*E* = 50,647
6	Group C-2: Solitary, larger SBC apart from joint line	12 mm	10.27 mm	6.79 mm		1,506,649	7,440,907	*N* = 12,726
7	Group C-3: Paired, larger SBCs model	12 mm, 12 mm	Ant: 7.22 mm Post: 7.55 mm	Ant: 7.08 mm Post: 6.43 mm	17.97 mm	1,518,584	7,495,331	*E* = 50,451

*SBC, subchondral bone cyst; FE, finite element; Ant, anterior; Post, posterior; b/w, between.*

### Von Mises Stress Distribution in Subchondral Bones

**FIGURE 3:**
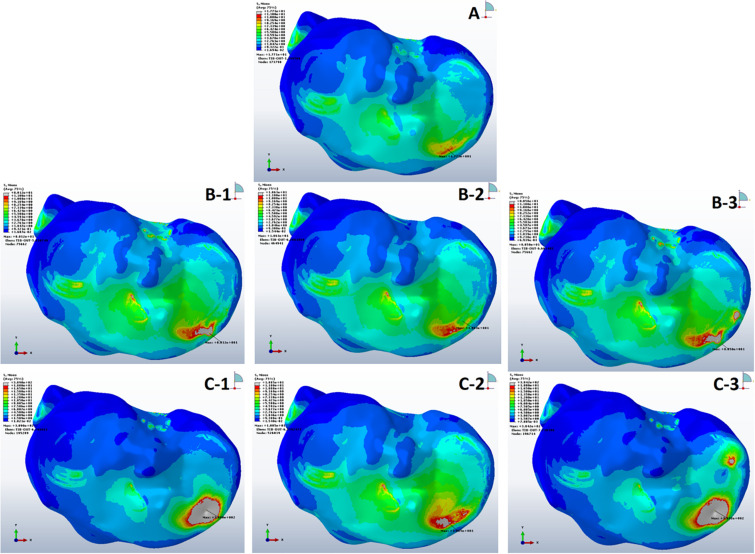
Von Mises stress distribution in subchondral bones during single-leg stance (750 N). **(A)** Non-cystic knee model. Groups B and C (represented by letters **B,C**) with the corresponding sub-groups 1, 2, and 3.

The mean VMS in subchondral bone of SBC models was higher than the non-cystic knee model (Figure 3). During single-leg stand condition, the non-cystic model showed the mean stress value of 9.35 ± 1.94 MPa, whereas in group B (smaller-sized SBC), the mean stress values were 37.71 ± 19.52, 9.93 ± 1.94, and 41.97 ± 20.77 MPa in sub-groups 1, 2, and 3, respectively. There was a significant increase (*p* < 0.001) in subchondral bone plate stress in all the sub-groups of group C. Further detail is given in Table 3.

**TABLE 3:** Statistical analysis of stress in subchondral and cancellous bone and cystic strains.

Variables	Groups	Sub- groups	Mean	Range	*Post hoc*-multiple comparisons	
					Mean difference	*p*-values*
Subchondral bone stress	A (Non-cystic)		9.35	7.78–17.71	−28.36* (A-B1) −0.59 (A-B2) −32.62* (A-B3)	0.044 (A-B1) 0.254 (A-B2) 0.026 (A-B3)
	B (4 mm cyst)	1	37.71	23.62–80.12	−114.18* (B1-C1)	0.000 (B1-C1)
		2	9.93	8.90–18.63	−2.16* (B2-C2)	0.000 (B2-C2)
		3	41.97	23.76–80.50	−131.62* (B3-C3)	0.000 (B3-C3)
	C (12 mm cyst)	1	151.89	81.02–389.05	142.54* (C1-A)	0.000 (C1-A)
		2	12.09	11.27–18.85	2.75* (C2-A)	0.000 (C2-A)
		3	173.58	114.51–384.23	164.24* (C3-A)	0.000 (C3-A)
Cancellous bone stress	A (Non-cystic)		5.27	5.01–5.64	−3.28* (A-B1) −1.25* (A-B2) −3.49* (A-B3)	(A-B1) (A-B2) 0.000 (A-B3)
	B (4 mm cyst)	1	8.56	7.93–9.04	−14.18* (B1-C1)	0.000 (B1-C1)
		2	6.52	6.14–7.33	−0.39* (B2-C2)	0.002 (B2-C2)
		3	8.76	8.59–9.04	−14.32* (B3-C3)	0.000 (B3-C3)
	C (12 mm cyst)	1	22.74	20.65–26.40	17.47* (C1-A)	0.000 (C1-A)
		2	6.92	6.47–8.54	1.65* (C2-A)	0.000 (C2-A)
		3	23.08	22.15–26.40	17.82* (C3-A)	0.000 (C3-A)
Strain analysis	A (Non-cystic)		0.0026	0.0003–0.003	−0.00476* (A-B1) −0.00237* (A-B2) −0.00498* (A-B3)	(A-B1) (A-B2) 0.000 (A-B3)
	B (4 mm cyst)	1	0.0073	0.007–0.008	−0.02057* (B1-C1)	0.000 (B1-C1)
		2	0.0049	0.004–0.01	−0.00146* (B2-C2)	0.000 (B2-C2)
		3	0.0075	0.007–0.008	−0.00498* (B3-C3)	0.000 (B3-C3)
	C (12 mm cyst)	1	0.0279	0.02–0.04	0.02533* (C1-A)	0.000 (C1-A)
		2	0.0064	0.005–0.007	0.00383* (C2-A)	0.000 (C2-A)
		3	0.0319	0.02–0.04	0.02934* (C3-A)	0.000 (C3-A)

*Mean difference significant at p < 0.05.

### Stress Analysis of Peri-Cystic Cancellous Bones

**FIGURE 4:**
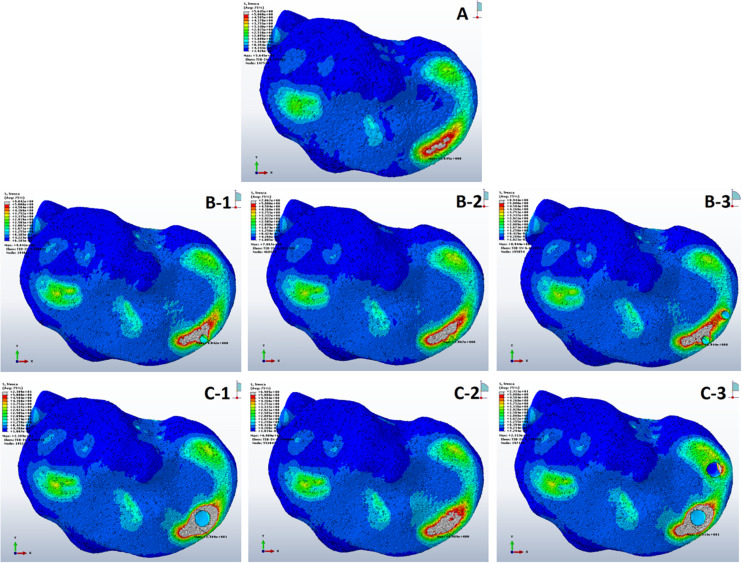
Stress distribution in peri-cystic cancellous bones. **(A)** Non-cystic knee model. Groups B and C (represented by letters **B,C**) with corresponding sub-groups1, 2 and 3.

Tresca stress analysis of the cancellous portion of medial tibia showed the least mean stress of 5.27 ± 0.14 MPa (5.01–5.64) in the anterior medial tibial plateau of non-cystic knee model. The bony defect caused by SBCs in group B: 8.56 ± 0.27, 6.52 ± 0.28, and 8.76 ± 0.14 MPa and group C: 22.74 ± 1.13, 6.92 ± 0.68, and 23.08 ± 0.86 MPa produced the statistically significant (*p* < 0.001) stress changes in the individual sub-groups, respectively. The detailed description of stress changes in all the three groups are depicted in Figure 4 and Table 3.

### Strain (*E*) Analysis of Cysts

**FIGURE 5:**
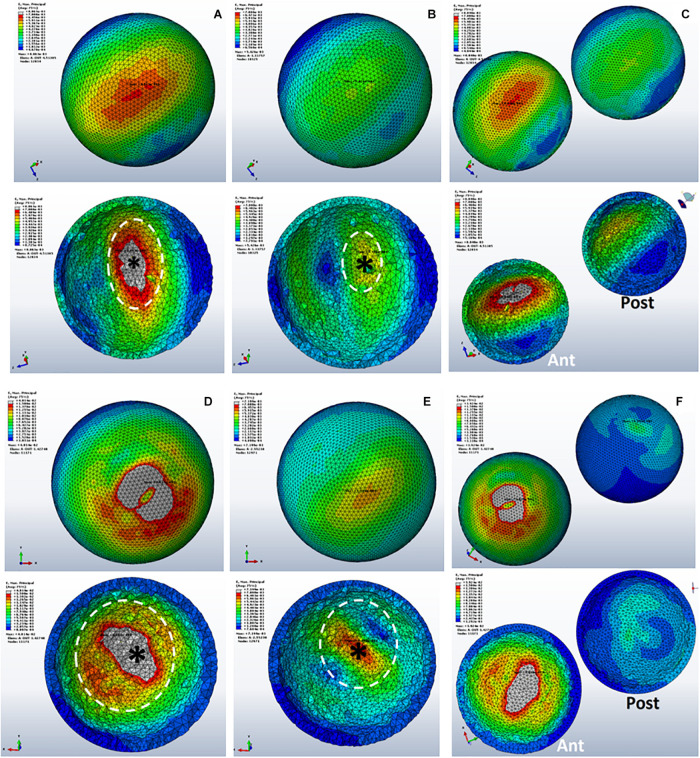
Strain distributions in SBCs. **(A–C)** Representing sub-groups 1, 2, and 3 of group B. Panels **(D–F)** showing sub-groups of group C with corresponding inner sections of cysts. The peak strain is pointed by “*,” and the dotted circle shows the direction of strain spread. SBCs, subchondral bone cysts.

On applying body weight, there resulted changes in the cysts. The resultant strain in all cysts is summarized in Figure 5 and Table 3. The inner section of individual cysts showed the peak areas of strain. The 12 mm-sized cyst in group C had highest mean strain value of 0.03 in the upper location near the joint line (sub-groups 1 and 3). Same-sized cyst at lower position showed nearly a 5 times less mean strain of 0.006. As the size of cysts increased from 4 to 12 mm, there was a prominent increase in strain (*p* < 0.001).

### Cartilage Shear Stress

**FIGURE 6:**
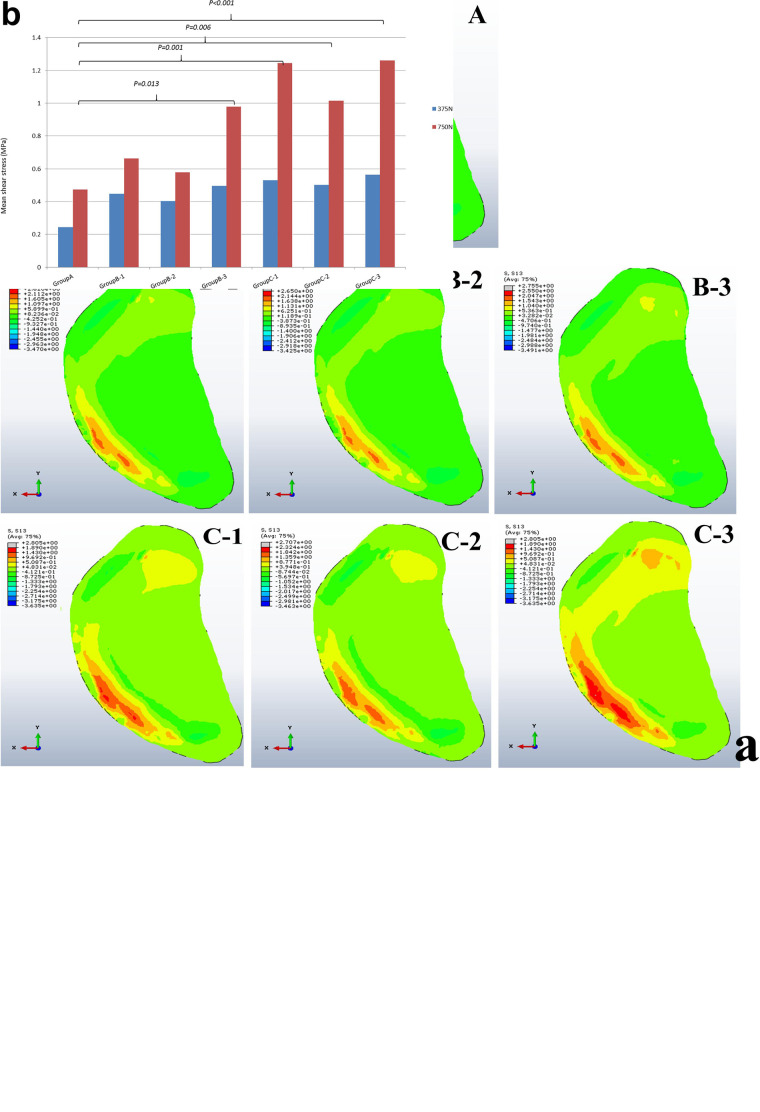
**(a)** Articular cartilage shear stress changes in groups A, B, and C with associated sub-groups 1, 2 and 3. **(b)** Graphical representation of mean cartilage stress during double and single-leg stance.

The mean shear stress analysis of medial tibial cartilage showed that the presence of a single small-sized cyst (sub-groups 1 and 2 of group B) with mean shear stress of 0.66 ± 0.56 and 0.58 ± 0.54 MPa was not significant as compared to non-cystic model (group A) (*p* = 0.374 and *p* = 0.590). However, the paired cysts of same size caused significant increase in stress changes (*p* = 0.011). On the other hand, the comparison between group A and group C (larger-sized SBCs) showed statistically significant increase in cartilage stress in all sub-groups: C-1, C-2, and C-3 (*p* = 0.001, *p* = 0.006, and *p* < 0.001) with mean stress values of 1.25 ± 0.69, 1.01 ± 0.54, and 1.26 ± 0.59 MPa than non-cystic knee model (0.47 ± 0.67 MPa), respectively. These focal increments in medial tibial cartilage are shown in Figure 6a and Table 4. Figure 6b depicts the increase in articular stress during double-leg and single-leg stands.

**TABLE 4:** Statistical analysis of cartilage stress in various groups.

Groups	Sub-groups	Mean	*Post hoc*-multiple comparisons		
			Mean difference	95%CI	*p* values*
A (Non-cystic)		0.47	−0.18778 (A-B1)	−0.61 to 0.23	0.374 (A-B1)
			−0.10338 (A-B2)	−0.49 to 0.28	0.590 (A-B2)
			−0.50510* (A-B3)	−0.89 to −0.12	0.011 (A-B3)
B (4 mm cyst)	B-1	0.66	−0.58320*(B1-C1)	−1.00 to −0.16	0.007 (B1-C1)
	B-2	0.58	−0.43698*(B2-C2)	−0.82 to −0.05	0.026 (B2-C2)
	B-3	0.98	−0.28146 (B3-C3)	−0.66 to 0.10	0.147 (B3-C3)
C (12 mm cyst)	C-1	1.25	0.77098*(C1-A)	0.35 to 1.19	0.001 (C1-A)
	C-2	1.01	0.54036* (C2-A)	0.17 to 0.92	0.006 (C2-A)
	C-3	1.26	0.78657* (C3-A)	0.40 to 1.17	0.000 (C3-A)

*Mean difference significant at p < 0.05.

## Discussion

According to our findings, on applying the tested loads (single- and double-leg stance) there was prominent increase in peri-cystic bone stress. We also noted significant increase (nearly 2 to 3 times) in mean cartilage stress in SBC models as compared to the non-cystic knee model. The mean shear stress of medial tibial cartilage in non-cystic model showed nearly uniform stress distribution (represented by letter “A” in Figure 6a), and the peak stress was in the antero-medial part of articular cartilage because in the standing position the medial compartment of the knee bears up to 2.2 times as much loading as the contrary lateral compartment (Johnson et al., 1980; D’Lima et al., 2007; Kutzner et al., 2010). However, SBCs increased the cancellous and subchondral bone stress, and there was a focal increase in the articular shear stress. For the cysts in group B, sub-group 3 affected the larger area in both the osseous and cartilaginous components. These altered areas of stress caused significant increase in cartilage stress. The small-sized solitary cyst (sub-groups 1 and 2 of group B) had higher mean cartilage stress than the non-cystic model but were statistically non-significant. On the other hand, there was a significant increase in focal cartilage stress (which may cause degeneration effect) caused by larger-sized SBCs (Table 4).

In a previous clinical study, the authors advocated that the presence of SBCs leads to degeneration and wasting of cartilage and also increases the risk of surgical knee replacements (Tanamas et al., 2010). Our findings provide the biomechanical bases of how SBCs of different parameters (size, numbers, and locations) can cause degenerative effect on cartilage (i.e., increased stress effect). These findings also strengthen the hypothesis proposed by Radin and Rose (1986) that the progression of articular degeneration is associated with perturbations in underlying bone.

The maximum strain was noted on the cyst surface facing the joint line. Inner sections of the cysts showed that the maximum strain was at the top of the cyst (represented by “**^∗^**” in Figure 5). As this part of the cyst was very near to articular cartilage, the most deteriorating effect of SBCs on cartilage was in this position. This intra-cystic focal effect in 12 mm-sized cysts (C-1 and C-3) was nearly 4 times greater than that of SBCs in sub-groups B-1 and B-3. Only a 1.4-fold increase in strain was produced by cysts at lower joint position in group B (B-2) than the corresponding cyst in group C (C-2). The strain spread was toward peripheral regions of the cysts (dotted circle in Figure 5), which means that cysts may expand toward their peripheral regions. SBCs located at the lower joint positions (sub-groups 2 of groups B and C) and SBCs located in posterior tibial halves demonstrated lower strain values than the same-sized SBCs at higher joint positions. This difference is clearly seen in Figures 8A,B, where the green line represents the strains in different groups.

**FIGURE 7:**
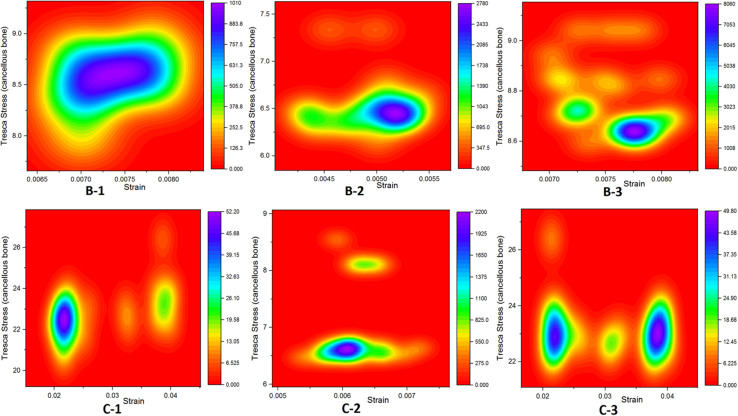
Graphical representation relationship between cancellous bone stress (bone resorption) and strain.

**FIGURE 8:**
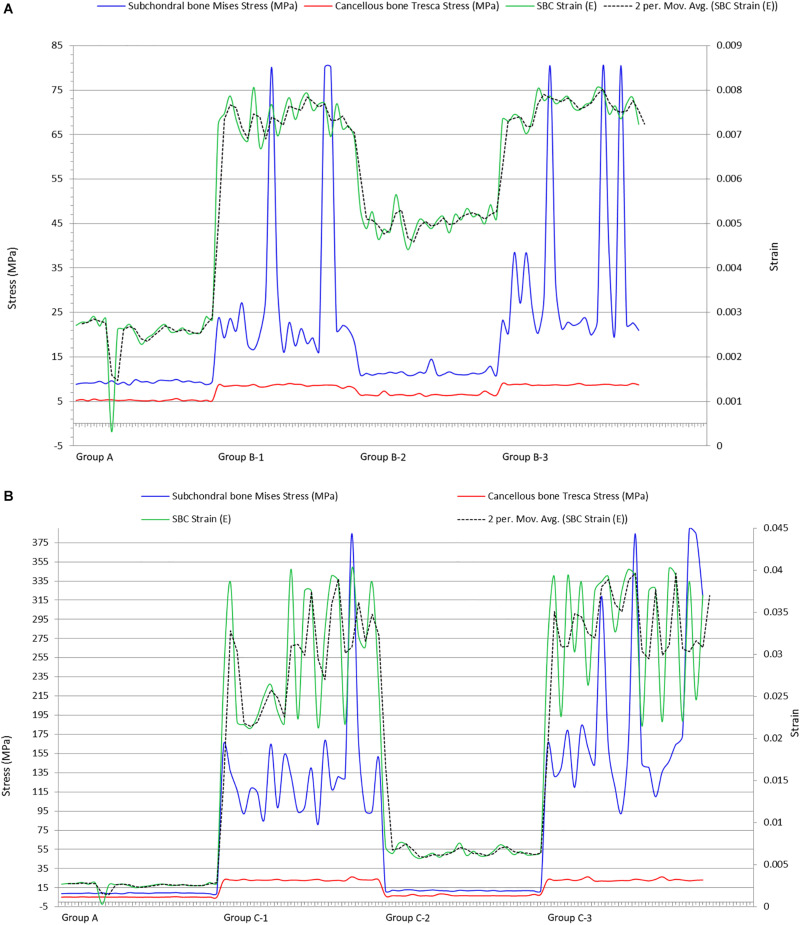
Plots showing impact of SBCs strain on subchondral and cancellous bones in **(A)** group B and **(B)** group C. SBCs, subchondral bone cysts.

Sabokbar et al. (2000) implied that elevated pressure in bones surrounding the SBCs could evoke a macrophage response within subchondral marrow and contribute to bone resorption and cyst enlargement. Our results showed 1.6-fold increase in peri-cystic cancellous bone stress in group B than the non-cystic model (group A). This stress effect was increased up to 4.5-fold in SBC model of 12 mm size (group C). Increased intra-osseous stress may result in boney destruction and subsequent SBC expansion. The stress produced by cysts in cancellous bone in relation to strain is depicted in Figure 7. A similar phenomenon of cysts expansion has been previously described in the hip joint (Ondrouch, 1963; Durr et al., 2004). Ondrouch (1963) carried out photo-elasticity experiments and concluded that cysts in OA may develop as a result of stress-induced bone resorption. Durr et al. (2004) studied the etiology of SBCs in a 2D FE hip model and advocated that micro-fractures in subchondral bone may be the initial step in the formation of SBCs. Our results showed that the mean VMS in subchondral bone of SBCs was increased up to 4∼4.5-fold in sub-groups 1 and 3 of group B and nearly 16∼18.5-fold higher in sub-groups 1 and 3 of group C as compared to the mean VMS in group A. In sub-group 2 of group B, only a slight increase in mean stress was observed (*p* = 0.254). But a significant increase in mean stress was observed in sub-group 2 of group C (*p* < 0.001). Therefore, the location of SBC in relation to the joint line is also important in case of small-sized cyst to produce significant stress changes (*p* = 0.044 vs. *p* = 0.254). The blue lines in Figure 8B shows tremendous increase in subchondral bone stress in sub-groups C-1 and C-3 in contrast to the subchondral bone stress in sub-groups B-1 and B-3 in Figure 8A. This signifies the size effect of SBCs (12 vs. 4 mm). The different location of same-sized cysts (B-1 vs. B-2 and C-1 vs. C-2) also produced different stress effects as represented by red and blue lines for cancellous and subchondral bones, respectively (Figures 8A,B). We should also keep in mind that the repeated or increased loading beyond the body weight (single-leg standing) may further deteriorate the condition (further increase stress effect).

## Conclusion

In conclusion, the bigger-sized SBCs in anterior and immediate position in the medial tibial cartilage produce increased focal stress effect in articular cartilage. Multiple cysts further deteriorate the condition by increased osseous stress effect and high tendency of peripheral cyst expansion.

## Data Availability Statement

The raw data supporting the conclusions of this article will be made available by the corresponding author on reasonable request.

## Ethics Statement

The studies involving human participants were reviewed and approved by the Institutional Review Board of First Affiliated Hospital of China Medical University. The patients/participants provided their written informed consent to participate in this study.

## Author Contributions

AA, ZH, YZ, YG, CT, MUN, YW, ZZhao, DL, ZZhan, HZ, and GL: study conception and design. AA, ZH, YZ, YG, CT, XW, MUN, YW, ZZhao, DL, ZZhan, and GL: acquisition of data. AA, ZH, YZ, YG, CT, XW, MUN, YW, ZZhan, DL, ZZhao, HZ, and GL: analysis and interpretation of data. All authors have revised and finally approved the manuscript.

## Conflict of Interest

The authors declare that the research was conducted in the absence of any commercial or financial relationships that could be construed as a potential conflict of interest.

## References

[B1] Bini F. Pica A. Marinozzi A. Marinozzi F. (2019). “Prediction of stress and strain patterns from load rearrangement in human osteoarthritic femur head: finite element study with the integration of muscular forces and friction contact,” in *New Developments on Computational Methods and Imaging in Biomechanics and Biomedical Engineering*, eds Fernandes P. R. Tavares J. M. R. S. (Cham: Springer), 49–64. 10.1007/978-3-030-23073-9_4

[B2] Bosiakov S. M. Alekseev D. V. Silberschmidt V. V. Shpileuski I. E. (2017). Effect of surgical defect localization on ultimate load-bearing capacity of human femur: finite-element energy-based assessment. *Proc. Struct. Integr.* 6 27–33. 10.1016/j.prostr.2017.11.005

[B3] Carrino J. A. Blum J. Parellada J. A. Schweitzer M. E. Morrison W. B. (2006). MRI of bone marrow edema-like signal in the pathogenesis of subchondral cysts. *Osteoarthr. Cartilage* 14 1081–1085. 10.1016/j.joca.2006.05.011 16806996

[B4] Chan P. M. B. Wen C. Yang W. C. Yan C. Chiu K. (2017). Is subchondral bone cyst formation in non-load-bearing region of osteoarthritic knee a vascular problem? *Med. Hypotheses.* 109 80–83. 10.1016/j.mehy.2017.09.027 29150301

[B5] Choi K. Kuhn J. L. Ciarelli M. J. Goldstein S. A. (1990). The elastic moduli of human subchondral, trabecular, and cortical bone tissue and the size-dependency of cortical bone modulus. *J. Biomech.* 23 1103–1113. 10.1016/0021-9290(90)90003-l 2277045

[B6] Crema M. D. Roemer F. W. Marra M. D. Niu J. Lynch J. A. Felson D. T. (2010). Contrast-enhanced MRI of subchondral cysts in patients with or at risk for knee osteoarthritis: the MOST study. *Eur. J. Radiol.* 75 e92–e96. 10.1016/j.ejrad.2009.08.009 PMC2891222 19767165

[B7] Crema M. D. Roemer F. W. Marra M. D. Niu J. Zhu Y. Lynch J. (2008). 373 MRI-detected bone marrow edema-like lesions are strongly associated with subchondral cysts in patients with or at risk for knee osteoarthritis: the MOST study. *Osteoarthr. Cartilage* 16:S160.

[B8] D’Lima D. D. Patil S. Steklov N. Chien S. Colwell C. W. Jr. (2007). In vivo knee moments and shear after total knee arthroplasty. *J. Biomech.* 40 S11–S17. 17462659 10.1016/j.jbiomech.2007.03.004

[B9] Donahue T. L. Hull M. L. Rashid M. M. Jacobs C. R. (2002). A finite element model of the human knee joint for the study of tibio-femoral contact. *J. Biomech. Eng.* 124 273–280. 10.1115/1.1470171 12071261

[B10] Durr H. D. Martin H. Pellengahr C. Schlemmer M. Maier M. Jansson V. (2004). The cause of subchondral bone cysts in osteoarthrosis: a finite element analysis. *Acta Orthop. Scand.* 75 554–558. 10.1080/00016470410001411 15513486

[B11] Freund E. (1940). The pathological significance of intra-articular pressure. *Edinburgh. Med. J.* 47 192–203. PMC5306552 29647906

[B12] Hopkins A. R. New A. M. Rodriguez-y-Baena F. Taylor M. (2010). Finite element analysis of unicompartmental knee arthroplasty. *Med. Eng. Phys.* 32 14–21. 10.1016/j.medengphy.2009.10.002 19897397

[B13] Innocenti B. Bilgen ÖF. Labey L. van Lenthe G. H. Sloten J. V. Catani F. (2014). Load sharing and ligament strains in balanced, overstuffed and understuffed UKA. A validated finite element analysis. *J. Arthroplasty* 29:1491. 10.1016/j.arth.2014.01.020 24581895

[B14] Innocenti B. Pianigiani S. Ramundo G. Thienpont E. (2016). Biomechanical effects of different varus and valgus alignments in medial unicompartmental knee arthroplasty. *J. Arthroplasty* 31 2685–2691. 10.1016/j.arth.2016.07.006 27519962

[B15] Innocenti B. Truyens E. Labey L. Wong P. Victor J. Bellemans J. (2009). Can mediolateral baseplate position andload sharing induce asymptomatic local bone resorption of the proximal tibia? A finite element study. *J. Orthop. Surg. Res.* 4:26. 10.1186/1749-799X-4-26 PMC2718929 19615054

[B16] Javaid M. K. Kiran A. Guermazi A. Kwoh C. K. Zaim S. Carbone L. (2012). Health ABC Study. Individual magnetic resonance imaging and radiographic features of knee osteoarthritis in subjects with unilateral knee pain: the health, aging, and body composition study. *Arthr. Rheum.* 64 3246–3255. 10.1002/art.34594 22736267 PMC3661953

[B17] Javaid M. K. Lynch J. A. Tolstykh I. Guermazi A. Roemer F. Aliabadi P. (2010). Pre-radiographic MRI findings are associated with onset of knee symptoms: the MOST study. *Osteoarthr. Cartilage* 18 323–328. 10.1016/j.joca.2009.11.002 19919856 PMC2990960

[B18] Johnson F. Leitl S. Waugh W. (1980). The distribution of load across the knee. A comparison of static and dynamic measurements. *J. Bone Joint Surg. Br.* 62 346–349. 10.1302/0301-620x.62b3.7410467 7410467

[B19] Kim H. J. Kim S. H. Chang S. H. (2011). Bio-mechanical analysis of a fractures tibia with composite bone plates according to the diaphyseal oblique fracture angle. *Compos. B Eng.* 42 666–674. 10.1016/j.compositesb.2011.02.009

[B20] Koh Y. G. Lee J. A. Kim Y. S. Kang K. T. (2019). Biomechanical influence of lateral meniscal allograft transplantation on knee joint mechanics during the gait cycle. *J. Orthop. Surg. Res.* 5:300. 10.1186/s13018-019-1347-y PMC6727551 31488183

[B21] Kornaat P. R. Bloem J. L. Ceulemans R. Y. Riyazi N. Rosendaal F. R. Nelissen R. G. (2006). Osteoarthritis of the knee: association between clinical features and MR imaging findings. *Radiology* 239 811–817. 10.1148/radiol.2393050253 16714463

[B22] Kutzner I. Heinlein B. Graichen F. Bende A. Rohlmann A. Halder A. (2010). Loading of the knee joint during activities of daily living measured in vivo in five subjects. *J. Biomech.* 43 2164–2173. 10.1016/j.jbiomech.2010.03.046 20537336

[B23] Landells J. W. (1953). The bone cysts of osteoarthritis. *J. Bone Joint Surg. Br.* 35-B 643–649. 10.1302/0301-620x.35b4.643 13108927

[B24] Marra M. D. Crema M. D. Chung M. Roemer F. W. Hunter D. J. Zaim S. (2008). MRI features of cystic lesions around the knee. *Knee* 15 423–438. 10.1016/j.knee.2008.04.009 18559292

[B25] McErlain D. D. Milner J. S. Ivanov T. G. Jencikova-Celerin L. Pollmann S. I. Holdsworth D. W. (2011). Subchondral cysts create increased intra-osseous stress in early knee OA: a finite element analysis using simulated lesions. *Bone* 48 639–646. 10.1016/j.bone.2010.11.010 21094285

[B26] Mesfar W. Shirazi-Adl A. (2005). Biomechanics of the knee joint in flexion under various quadriceps forces. *Knee* 12 424–434. 10.1016/j.knee.2005.03.004 15939592

[B27] Mow V. C. Ateshian G. A. Spilker R. L. (1993). Biomechanics of diarthrodial joints: a review of twenty years of progress. *J. Biomech. Eng.* 115 460–467. 10.1115/1.2895525 8302026

[B28] Ondrouch A. S. (1963). Cyst formation in osteoarthritis. *J. Bone Joint Surg. Br.* 45 755–760. 10.1302/0301-620x.45b4.755 14074329

[B29] Qiu T. X. Teo E. C. Yan Y. B. Lei W. (2011). Finite element modeling of a 3D coupled foot-boot model. *Med. Eng. Phys.* 33 1228–1233. 10.1016/j.medengphy.2011.05.012 21676642

[B30] Radin E. L. Rose R. M. (1986). Role of subchondral bone in the initiation and progression of cartilage damage. *Clin. Orthop. Relat. Res.* 213 34–40. 3780104

[B31] Raja Izaham R. M. Abdul Kadir M. R. Abdul Rashid A. H. Hossain M. G. Kamarul T. (2012). Finite element analysis of Puddu and Tomofix plate fixation for open wedge high tibial osteotomy. *Injury* 43 898–902. 10.1016/j.injury.2011.12.006 22204773

[B32] Raynauld J. P. Martel-Pelletier J. Berthiaume M. J. Abram F. Choquette D. Haraoui B. (2008). Correlation between bone lesion changes and cartilage volume loss in patients with osteoarthritis of the knee as assessed by quantitative magnetic resonance imaging over a 24-month period. *Ann. Rheum. Dis.* 67 683–688. 10.1136/ard.2007.073023 17728333

[B33] Rhaney K. Lamb D. W. (1955). The cysts of osteoarthritis of the hip; a radiological and pathological study. *J. Bone Joint Surg. Br.* 37-B 663–675. 13271499 10.1302/0301-620X.37B4.663

[B34] Roemer F. W. Eckstein F. Guermazi A. (2009). Magnetic resonance imaging-based semi quantitative and quantitative assessment in osteoarthritis. *Rheum. Dis. Clin. N. Am.* 35 521–555. 10.1016/j.rdc.2009.08.006 19931802

[B35] Sabokbar A. Crawford R. Murray D. W. Athanasou N. A. (2000). Macrophage-osteoclast differentiation and bone resorption in osteoarthrotic subchondral acetabular cysts. *Acta Orthop. Scand.* 71 2555–2561. 10.1080/000164700317411843 10919296

[B36] Sarrafpour B. El-Bacha C. Li Q. Zoellner H. (2019). Roles of functional strain and capsule compression on mandibular cyst expansion and cortication. *Arch. Oral Biol.* 98 1–8. 10.1016/j.archoralbio.2018.10.035 30419484

[B37] Takeda Y. Xerogeanes J. W. Livesay G. A. Fu F. H. Woo S. L. (1994). Biomechanical function of the human anterior cruciate ligament. *Arthroscopy* 10 140–147. 10.1016/b978-0-323-38962-4.00034-5 8003139

[B38] Tanamas S. K. Wluka A. E. Pelletier J. P. Martel-Pelletier J. Abram F. Wang Y. (2010). The association between subchondral bone cysts and tibial cartilage volume and risk of joint replacement in people with knee osteoarthritis: a longitudinal study. *Arthr. Res Ther.* 12:R58. 10.1186/ar2971 PMC2888209 20356405

[B39] Torres L. Dunlop D. D. Peterfy C. Guermazi A. Prasad P. Hayes K. W. (2006). The relationship between specific tissue lesions and pain severity in persons with knee osteoarthritis. *Osteoarthr. Cartilage* 14 1033–1040. 10.1016/j.joca.2006.03.015 16713310

[B40] Trad Z. Barkaoui A. Chafra M. Tavares J. M. R. (2018). *FEM Analysis of The Human Knee Joint: A Review.* Berlin: Springer International Publishing.

[B41] Wu H. Webber C. Fuentes C. O. Bensen R. Beattie K. Adachi J. D. (2007). Prevalence of knee abnormalities in patients with osteoarthritis and anterior cruciate ligament injury identified with peripheral magnetic resonance imaging: a pilot study. *Can. Assoc. Radiol. J.* 58 167–175. 17718300

