# Supplementary material for: Multiple Subchondral Bone Cysts Cause Deterioration of Articular Cartilage in Medial OA of Knee: A 3D Simulation Study
Source: Front Bioeng Biotechnol. 2020 Oct 6;8:573938. doi: 10.3389/fbioe.2020.573938 (PMC7583719; doi:10.3389/fbioe.2020.573938)
Supplement: Supplementary file 1 [file Table_1.DOCX]

**Supplementary Table 1. Patients demographical data.**

| S# | Age /Gender | Leg/Tibial plateau involved | Cyst diameter (mm) | No of cysts | Ant/post plateau | Distance from joint line* (mm) |
| --- | --- | --- | --- | --- | --- | --- |
| 1 | 57/F | Left/ Medial | 6.70, 3.5 | 2 | Ant+ Post | 4.5, 3.53 |
| 2 | 49/F | Right/ Medial | 8.9 | 1 | Post | 7.65 |
| 3 | 54/F | Left/ Medial | 12.0 | 1 | Ant | 7.22 |
| 4 | 63/F | Left/ Lateral | 7.9 | 1 | Ant | 2 |
| 5 | 51/M | Right/ Lateral | 11.35 | 1 | Ant | 7.75 |
| 6 | 52/F | Right/Medial | 4.0, 4.3 | 2 | Ant | 2.92, 2.98 |
| 7 | 56/F | Right/Lateral | 4.9 | 1 | Post | 5.2 |
| 8 | 61/F | Right/ Medial | 7.23 | 1 | Ant | 6.92 |
| 9 | 56/F | Left/ Medial | 12.0 | 1 | Ant | 10.27 |
| 10 | 63/M | Right/Lateral | 11.8 | 1 | Ant | 7.5 |
| 11 | 57/F | Right/ Medial | 11.15 | 1 | Post | 8.03 |
| 12 | 55/F | Left/ Lateral | 5.27 | 1 | Ant | 4.0 |
| 13 | 49/M | Left/ Medial | 9.38, 4.5 | 2 | Ant+ Post | 6.88, 3.27 |
| 14 | 62/F | Right/ Medial | 10.3 | 1 | Ant | 6.85 |
| 15 | 50/M | Right/Medial | 12.2, 12.05 | 2 | Ant+ Post | 7.20, 7.55 |
| 16 | 58/F | Right/ Lateral+Med | 7.52, 4.1 | 2 | Ant | 4.76, 3.17 |
| 17 | 45/M | Right/Medial | 4.0 | 1 | Ant | 4.54 |
| 18 | 50/F | Left/ Lateral | 6.77 | 1 | Ant | 5.78 |
| 19 | 50/F | Left/ Lateral | 7.20 | 1 | Ant | 4.7 |
| 20 | 55/M | Right/Medial | 4.0 | 1 | Post | 2.92 |
| 21 | 63/F | Right/ Medial | 7.7, 4.4 | 2 | Ant+ Post | 5.3, 4.8 |
| 22 | 62/F | Left/ Medial | 5.5 | 1 | Ant | 3.7 |

*Distance between cyst center and articular surface
